# Endoscopic removal of esophageal foreign body embedded in muscularis propria

**DOI:** 10.1055/a-2239-3296

**Published:** 2024-01-30

**Authors:** Lingzhi Qin, Haitao Shi, Xue Zhang, Peiqi Chen, Xin Liu, Jinhai Wang, Bin Qin

**Affiliations:** 1 117799 Department of Gastroenterology, Xiʼan Jiaotong University Second Affiliated Hospital, Xiʼan, China

**Fig. 1:**
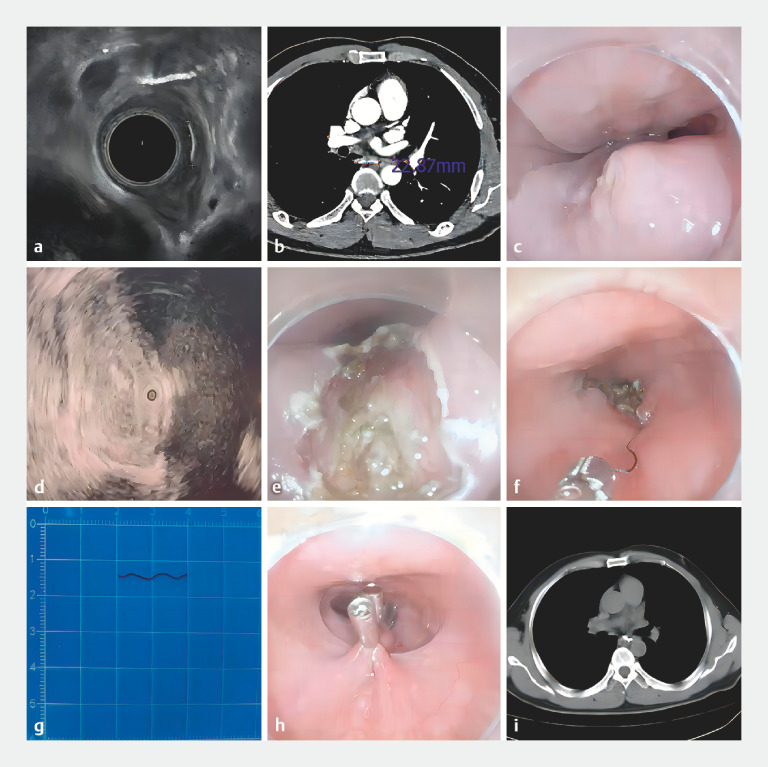
Illustration of the major steps involved in endoscopic removal of an esophageal foreign body embedded in the muscularis propria. Endoscopic removal of the entirely embedded esophagus-penetrating foreign body in a 53-year-old man presenting with a 3-week history of retrosternal pain. **a** Visualization of the foreign body under endoscopic ultrasonography. **b** Visualization of the foreign body under enhanced computed tomography. **c** Visualization of the granulation tissue protruding from the posterior esophageal mucosa, with a small amount of purulent discharge. **d** Localization of the foreign body with the guide of the ultrasound probe. **e,f** Myotomy, foreign body exposure, and extraction. **g** The foreign body was a curved metal wire, 20 mm in length. **h** Closure of the esophageal wound. **i** Postoperative computed tomography of the chest showed no residual foreign body left.

A 53-year-old man presented with a 3-week history of retrosternal pain after the ingestion of hard food. A computed tomography (CT) scan of the chest indicated a transverse high-density opacity in the middle of the esophagus, extending into the right mediastinum. Esophagoscopy showed edema of the mucosa and granulation tissue at the right posterior esophageal wall 31 cm from the incisors, surface erosion with a small amount of purulent discharge, and no foreign body found in the lumen. Ultrasonography suggested a hyperechoic area in the right posterior muscularis propria layer of the esophagus, connected to a hypoechoic lesion outside the esophagus ( Fig. 1 **a** ); imaging indicated an entirely embedded esophagus-penetrating foreign body and mediastinal abscess. A routine chest and enhanced CT scan revealed a high-density line, 22.9 mm in length, embedded in the middle esophagus and protruding into the mediastinum, with uneven soft tissue enhancement ( Fig. 1 **b** ). No vascular injury was detected.

Under general anesthesia, the patient’s esophagus was accessed with a flexible endoscope ( Video 1 ). Granulation tissue with erosion was observed at 31 cm from the incisors ( Fig. 1 **c** ). After exfoliating the mucosal and submucosal layers with a dual injection knife (MicroTech, Nanjing, China), the muscularis was exposed. However, the expected foreign body was not found on the surface of the muscularis. Under ultrasound guidance and following a hyperechoic linear region displayed in the muscular layer ( Fig. 1 **d** ), the incision proceeded until the muscularis propria was opened ( Fig. 1 **e** ), revealing a significantly thickened and fibrotic area. Forceps repeatedly attempted to grasp the suspected fibrous tissue. Fortunately, a linear curved metal wire was exposed and clamped before removing with the endoscope ( Fig. 1 **f** ).

Endoscopic removal of an entirely embedded esophagus-penetrating foreign body in a 53-year-old man presenting with a 3-week history of retrosternal pain.Video 1

The extracted iron wire was 20 mm long ( Fig. 1 **g** ). The incision was completely sealed using endoclips ( Fig. 1 **h** ). A nasogastric tube was inserted for the postoperative fasting period. Meglumine diatrizoate esophagography and chest CT 6 days later suggested a complete esophageal wall and no obvious fistula formation ( Fig. 1 **i** ).

Endoscopy_UCTN_Code_CCL_1AB_2AF

